# Machine-Washable Smart Textiles with Photothermal and Antibacterial Activities from Nanocomposite Fibers of Conjugated Polymer Nanoparticles and Polyacrylonitrile

**DOI:** 10.3390/polym11010016

**Published:** 2018-12-22

**Authors:** Dabin Lee, Jeong Seon Sang, Pil J. Yoo, Tae Joo Shin, Kyung Wha Oh, Juhyun Park

**Affiliations:** 1 School of Chemical Engineering and Materials Science, Institute of Energy Converting Soft Materials, Chung-Ang University, Seoul 06974, Korea; imdabin@naver.com; 2 Industry Academic-Cooperation Foundation, Chung-Ang University, Seoul 06974, Korea; sangjs88@naver.com; 3 School of Chemical Engineering and SKKU Advanced Institute of Nanotechnology (SAINT), Suwon 16419, Korea; pjyoo@skku.edu; 4 UNIST Central Research Facilities and School of Natural Science, Ulsan National Institute of Science and Technology (UNIST), Ulsan 44919, Korea; 5 Department of Fashion Design, College of Art, Chung-Ang University, Seoul 06974, Korea; kwhaoh@cau.ac.kr

**Keywords:** smart textiles, smart fabrics, conjugated polymer nanoparticles, photothermal conversion, antimicrobial fibers, polyacrylonitrile, nanocomposite fibers

## Abstract

Smart textiles based on conjugated polymers have been highlighted as promising fabrics that can intelligently respond to environmental stimuli based on the electrical properties of polymer semiconductors. However, there has been limited interest in the photothermal properties of conjugated polymers that can be applied to smart textiles. We prepared nanoparticles by assembling a conjugated polymer with a fatty acid via an emulsion process and nanocomposite fibers by distributing the conjugated polymer nanoparticles in a polyacrylonitrile matrix. We then fabricated the textiles using the fibers. The resulting fabrics based on nanocomposite fibers show a temperature increase to 50 °C in 10 min under white light irradiation because of efficient photothermal conversion by the conjugated polymer light harvester, while the temperature of a pristine polyacrylonitrile fabric increases to only 35 °C. In addition, excellent antimicrobial activity was confirmed by a 99.9% decrease in the populations of *Staphylococcus aureus* and *Escherichia coli* over 24 h because of the effect of the fatty acid in the nanocomposite films and fabrics. Furthermore, the fabric showed efficient durability after a laundry test, suggesting the usefulness of these smart textiles based on conjugated polymer nanoparticles for practical applications.

## 1. Introduction

Smart textiles that can sense environmental stimuli, and that can thermally, chemically, electrically, mechanically, or magnetically respond to stimuli, are increasingly attracting attention for applications in medical/health care, sports, fashion, and the military [1,2,3]. Conjugated polymers are among the most popular materials for smart textiles because their π-conjugated backbones endow a semiconducting property owing to intra- and intermolecular electron delocalization [4]. A variety of functionalities based on conjugated polymers have been incorporated into fabrics as electrodes, energy storage devices, photovoltaic cells, light-emitting diodes, or sensors. For example, electronic textiles [5,6,7,8,9], flexible lithium-ion batteries with a thin coating of microporous polymeric networks [10], electrochemical transistors [11], and full-color light-emitting fibers [12] have mainly utilized the electronic conductivity of conjugated polymers.

However, very few studies have utilized the other important property of the conjugated polymer, photothermal conversion. Conjugated polymers with both donor and acceptor structural units have been highlighted as efficient photosensitizers for photovoltaic [13] and photocatalytic [14] applications because of their excellent light harvesting ability in visible/near-infrared (NIR) light owing to narrow bandgaps. Furthermore, because of multiple energy decay processes after excitation by light absorption resulting from the donor–acceptor structures in molecular backbones, conjugated polymers often generate heat via radiationless decay rather than photoluminescence via direct charge recombination [15,16,17,18,19,20,21]. Thus, conjugated polymers with donor–acceptor structural units can absorb an enormous amount of energy over a broad spectral range and transform the energy to ultrasound waves or heat, thereby becoming the ideal candidate for photoacoustic imaging [22], photothermal therapy [23], and photodynamic therapy [24] to ablate cancer cells in theranostic applications. However, smart textiles that employ conjugated polymers as a heat generator via photothermal conversion after absorbing solar energy have not been widely studied. It is mainly because the focus of studies using conjugated polymers has been on the utilization of their semiconducting property for electrode materials [5,6,7,8,9]. Textiles with weavable electric heaters based on conjugated polymers [25] have only recently been introduced. 

Recently, we demonstrated that conjugated polymer nanoellipsoids assembled with a fatty acid could be composited with polyurethane via hydrogen bonding and that the resultant multifunctional nanocomposite films showed both photothermal conversion and antibacterial activities [26]. Surface carboxylic acids on the nanoellipsoids enabled them to be uniformly distributed in the polyurethane matrix via hydrogen bonding between the carboxylic acids and urethane units in the polyurethane. Nanocomposite films with only 1 wt % of nanoellipsoids reached a temperature higher than 40 °C in only 10 min of white light irradiation because of the photothermal conversion of the conjugated polymer with a narrow bandgap. They also eliminated 99.9% of the *Staphylococcus aureus (S. aureus)* and *Escherichia coli (E. coli)* from their surfaces because of the fatty acids. It is known that the acidity of the fatty acids can cause them to physically disturb the microbial cellular membrane structure, thereby increasing the fluidity and disorganization of the membrane, ultimately leaking intracellular components and finally disintegrating the cells [27,28,29]. This antibacterial activity is comparable to that of nanocomposite films with 1 wt % silver nanoparticles, which is among the most popular antibacterial materials [30,31]. The potential use of these multifunctional nanocomposite films for smart textiles remains a challenge because the fabrication of textiles based on the composites, and wash/wear resistance of the resulting textiles are required for practical applications, as demonstrated by the recent progress in electronic textiles based on conjugated polymers [5]. 

Here, we prepared nanocomposite polyacrylonitrile (PAN) fibers with conjugated polymer nanoparticles (CPNs) assembled with a fatty acid as a filler via a solution spinning process and fabricated textiles using the fibers. We show that the textiles have both photothermal and antibacterial activities and are practically applicable by examining their mechanical durability through washing in a laundry machine. CPNs of a conjugated polymer with donor–acceptor structural units and narrow bandgap, poly[2,6-(4,4-bis-(2-ethylhexyl)-4H-cyclopenta[2,1-b;3,4-b′]-dithiophene)-alt-4,7-(2,1,3-benzothiadiazole)] (PCPDTBT), assembled with octanoic acid (OA), were prepared using an emulsion process and dispersed in PAN fibers via hydrogen bonding during the solution spinning process. We show the mechanical durability together with the photothermal and antibacterial activities of the textiles based on nanocomposite PAN fibers. 

## 2. Experimental Section

### 2.1. Materials

**Scheme 1:**
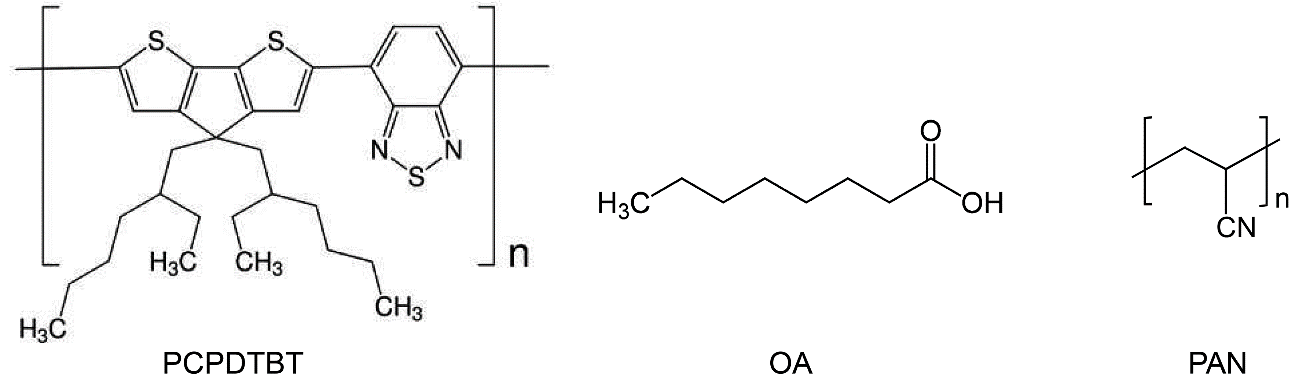
Chemical structures of poly[2,6-(4,4-bis-(2-ethylhexyl)-4H-cyclopenta[2,1-b;3,4-b′]-dithiophene)-alt-4,7-(2,1,3-benzothiadiazole)] (PCPDTBT), octanoic acid (OA), and nanocomposite polyacrylonitrile (PAN).

PCPDTBT (*M*_W_ = 34 kDa, PDI = 2.1, *M*_W_ on a repeat unit basis = 534.845 g/mol, One Materials, Inc., Quebec, Canada), OA (*M*_W_ = 144.21 g/mol, Sigma-Aldrich, St. Louis, MO, USA), PAN (powder, average *M*_w_ = 150,000 g/mol, Sigma-Aldrich), and organic solvents (Sigma-Aldrich) were used as received (Scheme 1).

### 2.2. CPN Preparation

CPNs were prepared by referring to our previous report [26]. In brief, 2.2 mL of OA solution (74 mg OA dissolved in 10 mL of chloroform) was added to 20 mL of dimethylsulfoxide (DMSO) under continuous stirring at 1500 rpm. After 15 min of stirring, 5 mL of PCPDTBT solution (1 mg of PCPDTBT dissolved in 10 mL of chloroform) was dropwise added and stirred for 1 h. The molar mixing ratio of the PCPDTBT on a repeat unit basis to OA was 1:12 (0.94 μM PCPDTBT:11.29 μM OA). The solution was further ultrasonicated for 5 min and heated at 80 °C for 3 h while stirred at 1500 rpm to completely evaporate the chloroform. 

### 2.3. Preparation of Nanocomposite Films, Fibers, and Textiles of PAN/CPNs 

The DMSO solution in which the CPNs were dispersed was poured into a DMSO solution with 18.0 wt % PAN and stirred at 1500 rpm. The weight percentage of the CPNs to PAN in the resulting solution was controlled at 0.75 wt %. To prepare the nanocomposite films, the solution was poured into a Petri dish and dried for 2 days at 25 °C followed by vacuum drying for 5 days at 80 °C. 

To prepare the nanocomposite fibers, PAN was dissolved in DMSO at 18 wt % and the DMSO solution with CPN dispersion was added into the PAN solution while stirred at 1500 rpm. Here, 0.75 wt % CPN was mixed with the PAN. Using a laboratory-scale wet-spinning machine with one coagulation bath (DMSO:deionized water = 50:50 wt %) and 11 washing baths (distilled boiling water), the mixture solution was extruded through a spinneret with 100 holes. The diameter of each hole was 0.1 mm. A total of 100 protofibers extruded at 25 m/min were combined into one strand, followed by coagulation in the first bath, washing in three consecutive baths, and natural drying with rolling fibers. As a control sample, PAN fibers were also prepared using 18 wt % PAN solution in DMSO. 

The nanocomposite fibers and PAN fibers were plied with commercial acrylic yarn (yarn count = 3/80 Nm) to ensure the mechanical stability of the resulting yarn because some of the filaments were broken seemingly due to inhomogeneous molecular assemblies during the precipitation in the non-solvents in the laboratory-scale wet-spinning process. The resulting yarn with six plies of the nanocomposite fibers and one ply of the commercial yarn was hand-knitted to produce 10 fabrics of a plain stitch (five from each of the yarns based on the nanocomposite fibers and PAN fibers, respectively) at a size of 5 cm × 5 cm. 

### 2.4. Characterization

UV-Visible absorption spectra of the PCPDTBT nanoparticles were obtained using an ultraviolet–visible (UV–vis) spectrometer (V-670, JASCO, Tokyo, Japan). ATR FT-IR spectra of PAN and PAN/CPN nanocomposite films were recorded using an FT-IR spectrometer (Nicolet 6700, Thermo Scientific, Waltham, MA, USA). Morphological observations of the nanostructures were conducted using a field-emission scanning electron microscope (FE-SEM, SIGMA, Carl Zeiss, Oberkochen, Germany) and a high-resolution transmission electron microscope (HR-TEM, JEM3010, JEOL, Akishima, Japan). Particle size analysis was performed by analyzing the SEM images of the CPNs using a GATAN Micrograph program. Structural analysis using two-dimensional (2D) grazing incidence X-ray diffraction (2D GIXD) was conducted at a synchrotron facility (6D UNIST-PAL beamline of PLS-II at Pohang Accelerator Laboratory, Pohang, Republic of Korea). To estimate the photothermal storage properties, the PU-CNP films were positioned at a distance of 20 cm from the solar simulator and heat generation images were recorded using an NIR camera (TG165, FLIR Systems, Wilsonville, OR, USA) every 10 s for 10 min under white light irradiation and for the next 10 min after turning off the light irradiation. Optical and fluorescent images of the composite fibers were recorded using a microscope (Olympus BX-51, Tokyo, Japan) with a Cy5 filter. Breaking forces of single filament nanocomposite fibers that were separated from the yarn were measured using an automatic single fiber test machine (FAVIMAT, Textechno, Moenchengladbach, Germany) at 5 mm/min testing speed and 25 mm gauge length. 

### 2.5. Antibacterial Test

The antibacterial properties of the blank (no films in dishes) films of PCPDTBT, OA, and PAN nanocomposite containing 0.75 wt % CPNs, fabrics based on PAN/CPN nanocomposite fibers before and after washing were monitored using three types of bacteria, *S. aureus* (ATCC 6538P, American Type Culture Collection, Manassas, VA, USA), *E. coli* (ATCC 8739), and Klebsiella pneumoniae (*K. pneumoniae*, ATCC 4352) by incubating the bacteria on films or fabrics in Petri dishes at 35 °C [26]. For film samples, the average concentrations of *S. aureus* and *E. coli* immediately after inoculating the cells on blank dishes (*M*_a_ in the colony forming unit/mL (CFU/mL)) and after 24-h incubation (*M*_b_) on the blank dishes were estimated as reference concentrations. On the other hand, the bacterial concentrations after incubation for 24 h on dishes with PCPDTBT, OA, and nanocomposite films of PAN containing 0.75 wt % CPNs (*M*_c_) were evaluated to estimate the antibiosis behaviors of the source materials and composite films. The source materials, PCPDTBT and OA, were coated on the surfaces of Petri dishes, and the proliferation of *S. aureus* and *E. coli* on the dishes were observed. For fabric samples, antibacterial activities were evaluated following a Korean Standard procedure (KS K0693). *S. aureus* and *K. pneumoniae* were incubated for 18 h on textiles based on PAN/CPN nanocomposite fibers before and after washing. To obtain reference concentrations for the fabric samples, bacteria were cultured on a textile based on cotton as a control.

(1) $$\mathrm{Antimicrobial}\;\mathrm{activity}\;(S)=\log\frac{M_{b}}{M_{c}}$$

(2) $$\mathrm{Degree}\;\mathrm{of}\;\mathrm{concentration}\;\mathrm{reduction}\;(\%)=\left[\frac{M_{b}-M_{c}}{M_{b}}\right]\times 100$$

### 2.6. Washing by Launder-Ometer

To mimic the variation in textile color by chemicals and friction during repeated machine-washing in daily use, textiles in our study were examined using an accelerated laundering test for a short time at a mildly elevated temperature. The conditions for the machine washing were set by following the test method 61-2010 of the American Association of Textile Chemists and Colorists (AATCC, Table 1). A standard detergent of the European Colorfastness Establishment (ECE-1) excluding sodium perborate, a color bleaching agent, regulated by ISO 105-C06:2010 or a neutral liquid detergent with a weaker chemical power than the standard detergent, was added to 1 L of the aqueous solution including the textiles. After washing, the textiles were removed and further rinsed twice with 100 mL of deionized water at 40 °C for 1 min, followed by natural drying for 1 day. The colorfastness of the textiles to washing was evaluated using KS K ISO 105-A02:2014 that includes nine grades of the colorfastness from 1, 1–2, 2, 2–3, 3, 3–4, 4, 4–5 to 5. 

## 3. Results and Discussion

### 3.1. Preparation of CPNs 

**Figure 1:**
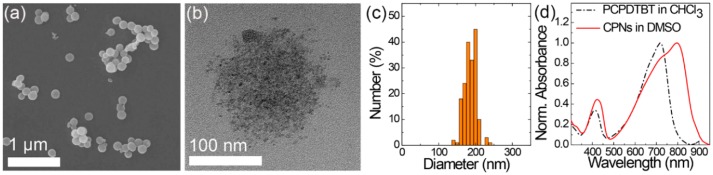
(**a**) SEM and (**b**) TEM images of PCPDTBT nanoparticles assembled with OA at a molar mixing ratio of PCPDTBT to OA of 1:12 and (**c**) particle size distribution. (**d**) Absorption spectra of the PCPDTBT dissolved in CHCl_3_ (reproduced from Ref. [26] with permission. Copyright 2018, Elsevier) and assembled into CPNs.

Spherical CPNs of PCPDTBT and OA formed in the DMSO (Figure 1a) via an emulsification process. A chloroform solution of PCPDTBT was dropwise added to a DMSO solution of OA with stirring, followed by formation of the emulsion of the chloroform solutions and removal of chloroform at an elevated temperature of 80 °C, a higher temperature than its boiling point. OA played the role of an emulsifier at the interface between the droplets of the chloroform solution and the DMSO medium because carboxylic acids in OA favor the polar medium of DMSO. Therefore, PCPDTBT and OA nanospheres were produced after removing the chloroform. The spherical shape of the CPNs differs from that of the nanoellipsoids that were produced when dimethyl formamide (DMF) was used instead of DMSO [26]. It is presumed that the intermolecular interactions between the DMF and carboxylic acids of OA enhanced the growth of the molecular assembly in one direction as shown in the nanowire assembly of PCPDTBT and octylbenzoic acids [32]. In this study, such intermolecular interactions between DMSO and OA seemed weak, thereby inducing the formation of spherical CPNs by minimizing the surface tension of the droplets. A TEM image of a CPN (Figure 1b) shows a spherical aggregate of PCPDTBT assemblies with OA. The average particle size of the CPNs obtained by analyzing the CPNs in the SEM images was 185.8 ± 16.7 nm (Figure 1c). The absorption spectrum of the CPNs (Figure 1d) shows that the absorption maximum of the PCPDTBT assembled in the CPNs is significantly red-shifted to 796 nm in comparison to that of the PCPDTBT dissolved in the chloroform (717 nm). The 79 nm of bathochromic shift in the absorption spectrum was attributed to the intermolecular electronic delocalization between the backbones of the PCPDTBT in the CPNs, as demonstrated in the films and PCPDTBT films and nanoparticles used for the photovoltaic cell and biomedical fields [33,34,35]. This result indicates that the PCPDTBT backbones are closely associated in CPNs, resulting in a prominent enhancement in NIR absorption. 

### 3.2. Characterization of CPNs, Fibers, and Films 

**Figure 2:**
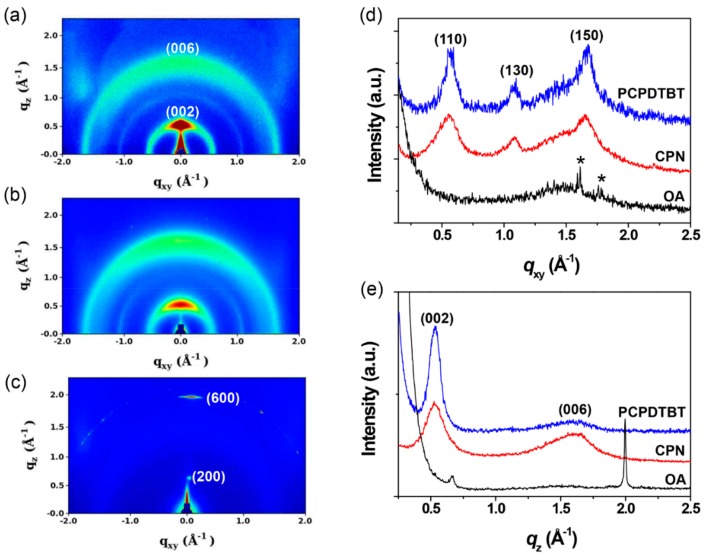
2D GIXD patterns of (**a**) PCPDTBT film, (**b**) CPNs, and (**c**) OA film, and corresponding (**d**) in-plane and (**e**) out-of-plane GIXD profiles. The asterisks (*) in (**d**) indicate diffraction peaks from the substrate.

The molecular assembly structure in the CPNs was examined using GIXD after drop casting and drying the CPN dispersion in the DMSO on silicon substrates. As control samples, chloroform solutions of PCPDTBT and OA were also drop-cast onto the substrates and films formed after drying were used for GIXD measurements. The 2D GIXD pattern of the CPNs (Figure 2a) does not show the characteristic peaks of the OA film (Figure 2c), but distinctly shows peaks similar to those of PCPDTBT film (Figure 2b). It is noticeable that the three PCPDTBT peaks shown in the in-plane line-cut at q = 0.570, 1.084, and 1.674 Å^−1^ (Figure 2d) correspond to the (110), (130), and (150) planes in an orthorhombic cell (*a* = 12.8 Å, *b* = 19.7 Å, *c* = 23.6 Å) of crystalline PCPDTBT, which is consistent with the previous reports [36,37]. In our previous study, PCPDTBT and OA were emulsified in dimethylformamide (DMF) and formed layered structures in which PCPDTBT chains were assembled in OA bilayers. It seemed that hydrogen bondings between OA and DMF enhanced the growth of molecular assemblies in the long axis, resulting in the formation of nanoellipsoids [26]. Our current study uses DMSO as the emulsion medium in which there are no strong hydrogen bondings between OA and DMSO. As a result, surface tension minimization overwhelms hydrogen bondings between OAs and π–π interactions between conjugated planes, thereby producing nanoparticles with the different assembly structure. The (150) plane at q = 1.674 Å^−1^ (*d* = 3.75 Å) correlates to the π–π stacking between the conjugated planes of the PCPDTBT. As shown in Figure 2, the position of those characteristic peaks of crystalline PCPDTBT are almost retained but peak widths are widened when the PCPDTBT molecules are assembled with OAs. This result can be interpreted with the formation of smaller nanocrystals with a wide distribution of crystal plane, where the conjugated backbones are closely associated with each other and the resultant intermolecular electron delocalization causes the bathochromic shift in the absorption spectrum. The OA shows two distinct peaks in a one-dimensional (1D) out-of-plane line cut of its 2D GIXD image at q = 0.664 (*d* = 9.46 Å) and 1.997 Å^−1^ (*d* = 3.14 Å). The *d-*spacings estimated from the two peaks are comparable to the length of the OA and size of the polar heads, indicating that carboxylic acids associated with hydrogen bonding in a film of OA contribute to the diffraction peaks. After being assembled with the PCPDTBT in the CPNs, these characteristic peaks of OA disappear, suggesting that the OA molecules are integrated with the PCPDTBT chains in the CPNs without forming a bilayer structure. Based on these GIXD data together with the TEM image in Figure 1b, it is suggested that CPNs with an average diameter of about 186 nm are actually aggregations of many tiny nanoparticles with a diameter less than 10 nm that might have a PCPDTBT/OA core-shell structure. 

**Figure 3:**
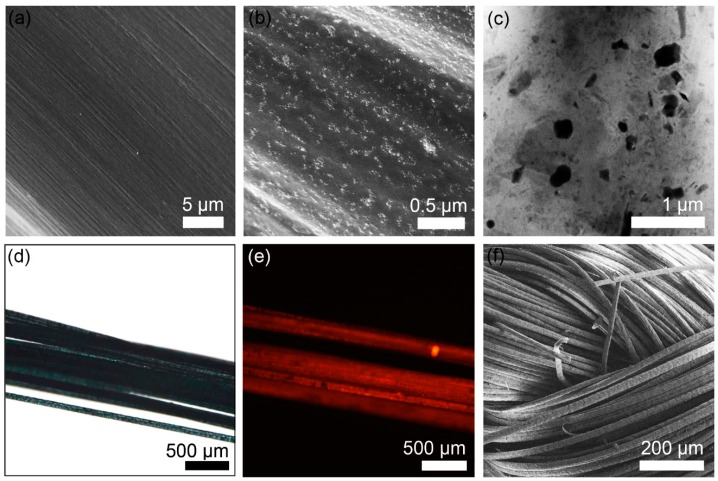
(**a**) SEM image of pristine PAN fiber. (**b**) SEM and (**c**) cross-sectional TEM images of a nanocomposite fiber of PAN with 0.75 wt % CPNs. (**d**) Optical and (**e**) fluorescent (excited at 650 nm) images of yarn based on nanocomposite fibers. (**f**) SEM image of fabric based on nanocomposite fibers.

CPNs (0.75 wt %) were successfully composited with PAN using a solution spinning process. When we examined the surfaces of the fibers based on pristine PAN and the composite of PAN and CPNs using SEM, we only observed lined scratches on the surface of the PAN fibers (Figure 3a) that were developed during extrusion of the fibers via nozzles. In comparison, CPNs were observed on the surface of the composite fibers as bright dots (Figure 3b). A cross-sectional TEM image (Figure 3c) also shows CPNs distributed in the matrix of PAN without serious aggregation. Incorporation of CPNs into yarns was also confirmed via optical and fluorescence images of yarns (Figure 3d and e, respectively). The red fluorescence originates from the CPNs distributed in the yarns. The yarns were used for textiles (Figure 3f). The diameter of the fibers estimated by analyzing 20 fibers in the SEM images was 19.84 ± 0.98 µm. 

**Figure 4:**
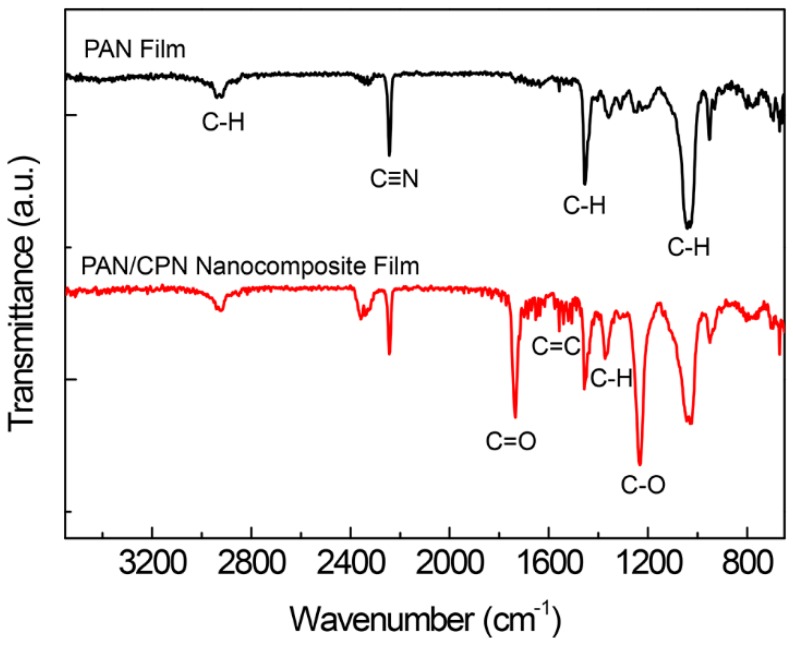
FT-IR spectra of a pristine PAN and a PAN/CPN nanocomposite films.

Possible interactions between the CPN fillers and the PAN matrix were monitored, measuring FT-IR spectra of a pristine PAN film and a nanocomposite film of PAN and CPNs (Figure 4). The pristine PAN film shows characteristic IR bands for asymmetric and symmetric C–H stretching at 2941.0 and 2919.7 cm^−1^, C≡N stretching at 2242.8 cm^−1^, C–H scissoring at 1454.1 cm^−1^, and C–H wagging at 1041.4 cm^−1^. After compositing the PAN with CPNs, the existence of OA and PCPDTBT were confirmed by IR bands of C=O stretching at 1733.7 cm^−1^, C=C stretching at around 1640 cm^−1^, methyl C–H rocking at 1373.0 cm^−1^, and C–O stretching at 1232.3 cm^−1^. There were no significant shifts of IR bands upon compositing, indicating that strong intermolecular interactions such as hydrogen bondings do not exist between nitrile groups in PAN and carboxylic acid groups in OA. The IR band of C≡N in the spectrum of the nanocomposite film was at the same wavenumber of 2242.8 cm^−1^ and that of C=O was positioned at the wavenumber of non-hydrogen bonded carbonyl while that of hydrogen-bonded carbonyl appeared at 1700 cm^−1^ [26]. However, the distribution of CPNs in the PAN matrix without aggregation shown in the cross-sectional TEM image (Figure 3c) suggests that there might be favorable interactions between the PAN matrix and CPN fillers such as dipole–dipole interactions, which are weaker than hydrogen bonds. 

### 3.3. Characterization of Antibacterial Activity

**Figure 5:**
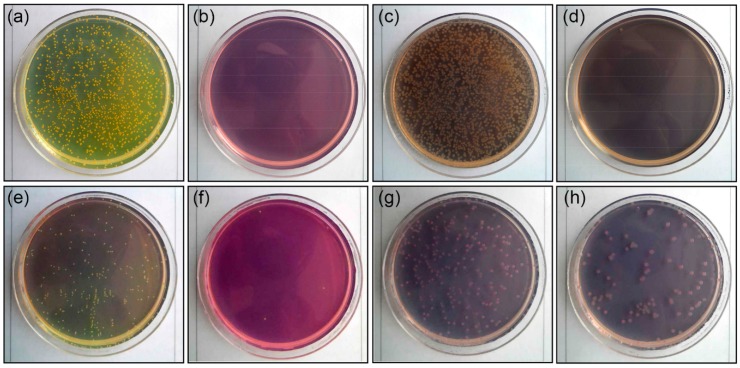
Antibacterial activity analysis *of S. aureus* for (**a**) blank and (**b**) nanocomposite films of PAN and CPNs at 0.75 wt %, and *E. coli* for (**c**) blank and (**d**) nanocomposite films of PAN and CPNs at 0.75 wt %. That of *S. aureus* (**e**) before and (**f**) after washing, and *K. pneumoniae* (**g**) before and (**h**) after washing fabrics based on PAN/CPN nanocomposite fibers.

Antibacterial activities were first examined for films of PCPDTBT, OA, and nanocomposites coated on Petri dishes and compared to the blank samples. Representative gram-positive and gram-negative bacteria, *S. aureus* and *E. coli*, respectively, were cultured on surfaces of blank Petri dishes and films. After 24 h incubation at 35 °C, 99.9% of the *S. aureus* and *E. coli* were eliminated on the OA and PAN/CPN nanocomposite film surfaces, showing the antibacterial activity above 6.0 (Table 2 and Figure 5), which was an excellent antibacterial effect comparable to that of silver nanoparticles [31,38]. Pristine PCPDTBT films coated on Petri dishes also showed significantly high antibacterial activities (2.3–2.5), eliminating bacteria over 99%. These results indicate that the antibacterial activity of the nanocomposite films originates from both PCPDTBT and OA. Then, antibacterial activities of fabrics based on PAN/CPN nanocomposite fibers were investigated before and after washing by incubating *S. aureus* and *K. pneumonia* (a gram-negative bacteria) on the fabrics for 18 h. The reduction of bacteria was over 99% for all fabrics, showing that the antibacterial activity was not affected by laundry washing. 

### 3.4. Influence of Washing on Photothermal and Mechanical Properties

**Figure 6:**
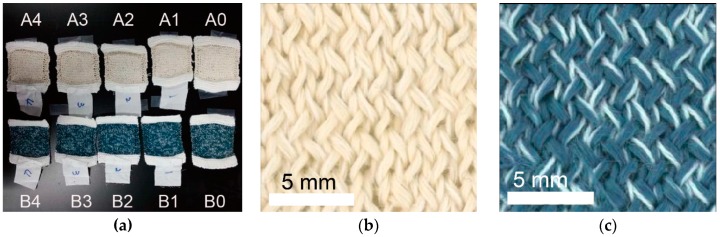
(**a**) An optical image of fabrics prepared from yarns based on PAN (A0‒A4) and PAN/CPN nanocomposite fibers (B0‒B4), and those of fabrics after washing, based on (**b**) PAN and (**c**) PAN/CPN nanocomposite fibers.

The durability of textiles during washing in a laundry machine was investigated for 10 textiles under five different conditions based on pristine PAN fibers (A set) and nanocomposite fibers (B set), as summarized in Table 1. The textiles were washed under four different conditions: (1) Standard chemical and physical condition, (2) weak chemical and standard physical condition, (3) no chemical and standard physical condition, and (4) weak chemical and no physical condition, and compared to the textile without washing (condition 0). Textiles before (A0 and B0) and after washing (A1–4 and B1–4) are shown in Figure 6a. The color fastness rating of all washed textiles was 4–5, the second highest rating among the 9 ratings from 1 to 5, and did not significantly deteriorate compared to that of the textile without washing. Representative optical images of the textiles based on PAN fibers and nanocomposite fibers after washing are shown in Figure 6b and 6c, respectively. Because the dark blue color of the textiles based on nanocomposite fibers originates from the CPNs that mainly absorb green and red light (Figure 1d), the color fastness rating of the textiles indicates that the CPNs are stable in the nanocomposite fibers under the influence of the chemical and physical forces of detergents and laundry machines, respectively. 

**Figure 7:**
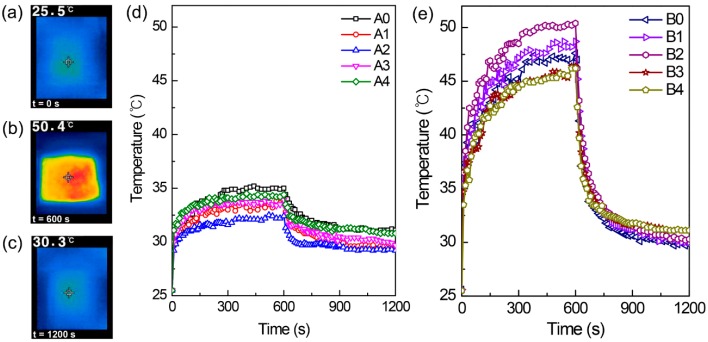
Infrared thermal images of B2 under white light irradiation at (**a**) t = 0 s and (**b**) t = 600 s, and (**c**) 600 s after turning off the light. The photothermal response of the textiles fabricated using (**d**) PAN and (**e**) PAN/CPN nanocomposite fibers at 0.75 wt % of CPNs under white light irradiation for the first 600 s, followed by 600 s after turning off the light.

The photothermal increase in textile temperatures was monitored before and after washing the textiles under white light irradiation using a solar simulator (Figure 7). The representative NIR images of the textiles before irradiating the white light, after 10 min of white light irradiation, and after another 10 min turning off the light are shown in Figure 7a, b, and c, respectively. The data plot in Figure 7d,e measured every 10 s shows no clear trend in the photothermal temperature increase depending on the washing conditions. For example, the textile based on the pristine PAN fibers without washing (A0) shows the best photothermal increase in comparison to the textiles with washing (Figure 7d), which seems reasonable, while the textiles based on nanocomposite fibers with washing under harsh conditions (B1 and B2) show a better photothermal performance than the textile without washing (B0) (Figure 7e). However, the increased temperatures in the plateau region after 6 min of light irradiation were 44 °C at a minimum for the textiles based on the nanocomposite fibers and 35 °C at a maximum for the textiles based on the pristine PAN fibers. These results clearly show the effectiveness of CPN fillers composited in the PAN matrix in increasing the photothermal property of the textiles.

**Figure 8:**
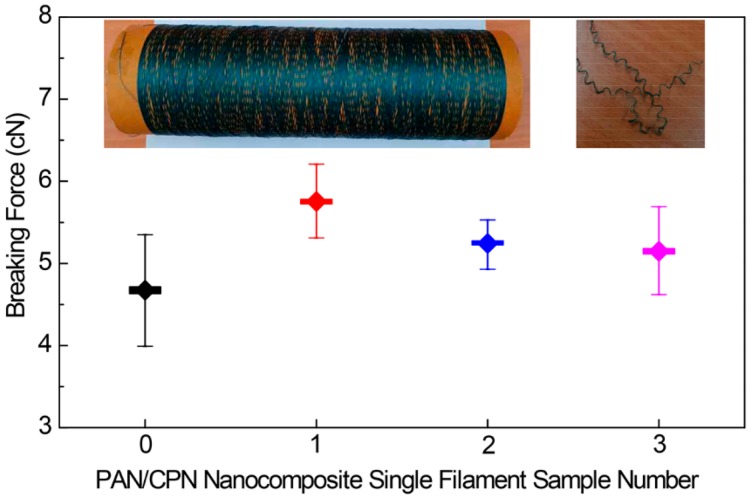
Breaking forces measured by single filament tensile experiments of pristine PAN fibers (filament 0), nanocomposite fibers before knitting the textiles (filament 1, left inset image), after knitting followed by deknitting without washing (filament 2), and after washing in a laundry machine followed by deknitting (filament 3, right inset image).

Durability of the mechanical properties before and after the washing textiles was examined by measuring the breaking force of four single filaments that were separated from the yarns of the pristine PAN fibers (Sample 0), the PAN/CPN nanocomposite fibers before knitting the textiles (Sample 1, the left inset image in Figure 8), deknitting from textiles before washing (Sample 2), and after washing in the laundry machine (Sample 3, the right inset image in Figure 8). The breaking forces of Samples 0, 1, 2, and 3 averaged from 17–20 filaments for each sample were 4.68 ± 0.36, 5.74 ± 0.25, 5.27 ± 0.17, and 5.13 ± 0.29 cN, respectively, which could be converted to tensile strengths of 151.28, 185.78, 170.54, and 165.94 MPa, respectively, for the fibers with an average diameter of 19.84 µm. These values for Samples 2 and 3 are 91.8% and 89.4% of the value of Sample 1, showing that an approximately 10% loss occurs in the mechanical properties during the knitting and deknitting processes of the textiles. However, the loss of mechanical properties by washing with a detergent in a laundry machine is within the range of the standard deviation, showing the durability of the textiles during machine washing.

## 4. Conclusions

We prepared nanoparticles of a donor–acceptor type conjugated polymer, PCPDTBT, by assembling them with a fatty acid, OA, using an emulsification process. Spherical nanoparticles of PCPDTBT/OA assemblies with an average diameter of about 186 nm had surface carboxylic acids and were successfully dispersed in the PAN fibers and on their surfaces via a solution spinning process. The resulting yarns of the nanocomposite fibers could be knitted to textiles. Under white light irradiation, the textiles showed an excellent photothermal temperature from 25 °C up to 55 °C in 10 min at maximum while the temperature of the textile based on pristine PAN fibers only increased to 35 °C. In addition, films of the nanocomposite bearing the conjugated polymer nanoparticles assembled with the fatty acid showed excellent antibacterial activity comparable to Ag nanoparticles. Most importantly, the textiles knitted using the nanocomposite fibers showed mechanical and antibacterial durability, and a strong color fastness during washing in a laundry machine, strongly suggesting their practical use as smart textiles. 

## Figures and Tables

**Table 1:** Conditions set by following the Association of Textile Chemists and Colorists (AATCC) test method for washing in a laundry machine for textiles knitted using yarns based on pristine PAN fibers and nanocomposite fibers of PAN and conjugated polymer nanoparticles (CPNs).

Laundry Condition	Temperature (°C)	Time(min)	Detergent	Launder-Ometer
0	–	–	No washing	–
1	40 ± 2	30	0.4 wt % standard	Used
2	40 ± 2	30	0.4 wt % neutral	Used
3	40 ± 2	30	Not used	Used
4	40 ± 2	30	0.4 wt % neutral	Not Used

**Table 2:** Antibacterial activity and reduction in bacterial concentrations on the surfaces of Petri dishes coated with PCPDTBT, OA, and PCPDTBT/CPN nanocomposite films, and on fabrics before and after washing.

Sample	Bacterium	M_a_ (CFU/mL) ^1^	M_b_ (CFU/mL)	M_c_ (CFU/mL)	Antibacterial Activity (S)	Reduction of Bacteria (%)
PCPDTBT Film	*S. aureus*	2.3 × 10^5^	1.1 × 10^7^	5.5 × 10^4^	2.3	99.5
PCPDTBT Film	*E. coli*	2.3 × 10^5^	1.2 × 10^7^	3.6 × 10^4^	2.5	99.7
OA Film	*S. aureus*	2.3 × 10^5^	1.1 × 10^7^	<10	6	99.9
OA Film	*E. coli*	2.3 × 10^5^	1.2 × 10^7^	<10	6.1	99.9
PAN/CPN Film	*S. aureus*	2.4 × 10^5^	1.2 × 10^7^	<10	6.1	99.9
PAN/CPN Film	*E. coli*	2.3 × 10^5^	1.3 × 10^7^	<10	6.1	99.9
Fabric, No Wash	*S. aureus*	1.4 × 10^5^	6.7 × 10^6^	5.4 × 10^4^	2.1	99.2
Fabric, No Wash	*K. pneumoniae*	1.6 × 10^5^	8.2 × 10^6^	2.1 × 10^4^	2.6	99.7
Fabric, Washed	*S. aureus*	1.4 × 10^5^	6.7 × 10^6^	1.3 × 10^4^	2.7	99.8
Fabric, Washed	*K. pneumoniae*	1.6 × 10^5^	8.2 × 10^6^	4.9 × 10^4^	2.2	99.4

^1^ Bacterial Concentration (CFU/mL): Numbers of colony-forming units per unit volume.
